# Comparative analysis of anal sphincter-preserving surgical techniques in ultra-low rectal cancer

**DOI:** 10.3389/fgstr.2026.1716349

**Published:** 2026-05-11

**Authors:** Hongjun Yuan, Jun Bu, Peng Zhang

**Affiliations:** Department of General Surgery, Sichuan University Affiliated Chengdu Second People’s Hospital, Chengdu, China

**Keywords:** anal function, anal sphincter-preserving, LARS, low anterior resection syndrome, ultra-low rectal cancer

## Abstract

Ultra-low rectal cancer refers to a tumor situated very close to the anal verge, typically within 3 cm from the proximal end of the anal dentate line. Its location creates significant challenges for achieving clear surgical margins necessary to achieve local tumor control while preserving sphincter function to minimize the risk of developing fecal incontinence and other complications.This review summarizes six sphincter-preserving techniques for ultra-low rectal cancer: local excision, low anterior resection(LAR, with or without prophylactic ileostomy or ileal stent), intersphincteric resection (ISR), modified Bacon and Parks procedures, transanal total mesorectal excision (TaTME), and Natural Orifice Specimen Extraction Surgery with Precision Functional Sphincter-Preserving Surgery(NOSES-PPS). While most approaches achieve satisfactory oncological outcomes, postoperative anal function remains a major concern. Preliminary evidence suggests NOSES-PPS may offer benefits in preserving anal function, though current studies are limited by small sample sizes and lack of large-scale trials. Optimal outcomes depend on patient selection, surgical expertise, and perioperative management.

## 1 Introduction

Ultra-low rectal cancer is commonly defined as a malignant tumor located within 3 cm of the anal dentate line. Traditionally, abdominoperineal resection (APR) represents the standard approach to removing these tumors and assessing any tumor involvement within the mesorectum. However, although this surgical procedure provides satisfactory local tumor control, it often necessitates the removal of the anal sphincter, thereby causing considerable physical and psychological morbidity. Advances in surgical techniques and perioperative management have facilitated the development and adoption of sphincter-preserving procedures for selected patients. Nowadays, low rectal cancer surgery aims to excise the tumor with an adequate circumferential resection margin while maintaining adequate postoperative anal sphincter function whenever feasible. However, complications following anal sphincter-preserving surgery are still common. One of the most common complications after anal sphincter–preserving surgery is postoperative low anterior resection syndrome (LARS). This condition, marked by frequent bowel movements, incontinence, urgency, and irregular defecation, can profoundly impair patients’ quality of life. LARS may lead to malnutrition, perianal skin problems, sleep disturbances, and psychological or social difficulties, including anxiety, depression, and limitations in daily activities or work. While ensuring complete tumor resection, maximizing the preservation of anal function, and reducing the incidence of LARS should also be considered as key criteria when selecting the optimal surgical procedure for ultra-low rectal cancer.

Therefore, in this literature review, we aimed to compare the indications, advantages, and limitations of different surgical procedures used to excise lower rectal tumors.

## 2 Methods

This review retrieved literature from several electronic databases, including PubMed, MEDLINE, Web of Science, and Embase, using keywords such as “ultra-low rectal cancer”, “anal sphincter-preserving”, “low anterior resection syndrome”, “intersphincteric resection”, “TaTME”, and “NOSES”. The inclusion criteria were: (1) studies reporting on surgical techniques for ultra-low rectal cancer (tumor located 1–3 cm from the dentate line); (2) clinical T1–T2 staging(or downstaged to T1–T2 after neoadjuvant therapy), recognizing that local excision is appropriate for selected T1 tumors, while more extensive sphincter-preserving resections (LAR, ISR, TaTME, NOSES-PPS, etc.) are typically indicated for T2 tumors or T1 tumors with adverse features such as poor differentiation or lymphovascular invasion; (3) tumor size <3 cm or involving ≤1/3 of bowel circumference; and (4) studies reporting postoperative anal function outcomes. Exclusion criteria were: (1) preexisting significant anal dysfunction; (2) distant metastasis; and (3) case reports or series with fewer than 5 patients.

Two independent reviewers screened titles, abstracts, and full texts, and extracted data on study characteristics, surgical technique, patient selection, and functional outcomes (including LARS scores, anastomotic leakage, and complication rates). Due to heterogeneity in study designs and outcome measures, a narrative synthesis was conducted to compare surgical techniques based on their indications, technical features, functional outcomes, and complication profiles.

## 3 Results

The literature search yielded 280 articles. A total of six surgical techniques for sphincter preservation in ultra-low rectal cancer were identified: local excision, low anterior resection (LAR) with prophylactic ileostomy or ileal stent, intersphincteric resection (ISR), modified Bacon and Parks procedures, transanal total mesorectal excision (TaTME), and natural orifice specimen extraction surgery with precision functional sphincter preservation (NOSES-PPS). To facilitate comparison across techniques, each procedure is described below with consistent reporting of indications, technical details, functional outcomes (including LARS incidence), and key advantages and limitations. A summary of comparative data is presented in **Table 1**.

**Table 1:** Comparison of sphincter-preserving surgical techniques for ultra-low rectal cancer.

Surgical Technique	Indications	LARS Incidence	Anastomotic Leakage Rate
Local Excision	T1, well-differentiated, <3 cm	Not applicable	Not applicable
LAR ± Ileostomy/Stent	>1 cm above dentate line	30–50%	15–28% without diversion
ISR	Tumor at or below levator ani	Higher than LAR	10–20%
Modified Bacon	Within 2 cm of anus	Higher than LAR	Near 0%
Modified Parks	Within 2 cm of anus	Higher than LAR	Higher than Bacon
TaTME	Low rectal cancer, narrow pelvis	Comparable to LAR	6.4–17.0%
NOSES-PPS	Ultra-low rectal cancer, <3 cm	Preliminary evidence suggests possibly lower than LAR	Preliminary evidence suggests possibly lower (12% overall complications)

### 3.1 Aim of sphincter-preserving surgery

The primary goal of sphincter-preserving surgery for ultra-low rectal cancer is to achieve radical oncological resection while maintaining postoperative anal function and overall quality of life. Optimal outcomes are achieved by preserving sphincter integrity and anorectal mucosa, including the dentate line, whenever feasible. For early-stage, small, well-differentiated tumors, a distal resection margin of 0.5 to 1 cm may be considered sufficient, provided that intraoperative frozen section analysis confirms negative margins. Additionally, dissection of the pericolic and intermediate node (D2) should also be performed routinely to reduce the risk of metastasis.

### 3.2 Surgical indications

A review of the current literature indicates that sphincter-preserving surgery for ultra-low rectal cancer is appropriate when certain oncological and functional criteria are satisfied. These include: (1) an inferior tumor margin located at least 1 cm above the dentate line; (2) a histology of moderately to well-differentiated adenocarcinoma, with clinical T1–T2 staging or effective downstaging to T1–T2 following neoadjuvant therapy; (3) preserved preoperative sphincter function; and (4) a tumor diameter of 3 cm or less or involvement of no more than one-third of the bowel circumference. However, depending on the clinical presentation, different surgical approaches may be required.

### 3.3 Types of anal sphincter preservation surgeries for ultra-low rectal cancer

The literature search revealed 6 different sphincter-preserving surgical approaches for ultra-low rectal cancer, including local excision techniques, low anterior resection (LAR) with prophylactic ileostomy or ileal stent, intersphincteric resection (ISR), modified Bacon and Parks procedures, transanal total mesorectal excision (TaTME), and natural orifice specimen extraction surgery with precision functional sphincter preservation (NOSES-PPS). These techniques vary widely in their indications, technical complexity, and impact on postoperative anal sphincter function.

#### 3.3.1 Local tumor excision

Local tumor excision can be performed using endoscopic resection, transanal minimally invasive surgery (TAMIS), or transanal endoscopic microsurgery (TEM) (1). Endoscopic resection is generally reserved for very superficial lesions and is performed entirely through the rectal lumen. TAMIS provides a minimally invasive transanal approach with improved visualization and greater instrument flexibility, allowing more precise excision of deeper lesions. TEM offers a platform for full-thickness excision under magnified endoscopic guidance for carefully targeted tumor removal. Both TAMIS and TEM are effective transanal platforms for local excision of selected early-stage rectal cancers; the choice between them depends on surgeon expertise, equipment availability, and tumor characteristics. While all three techniques facilitate safe resection of small tumors with minimal trauma and rapid recovery, which is particularly advantageous for elderly patients or those with compromised cardiopulmonary function, none of these endoscopic techniques can be used to assess regional mesorectal nodal involvement. Therefore, thorough preoperative imaging staging using magnetic resonance imaging (MRI) and endorectal ultrasound is essential to accurately evaluate tumor depth and nodal status before selecting the most appropriate approach.

#### 3.3.2 LAR with or without prophylactic ileostomy or ileal stent placement

Laparoscopic or open LAR is currently the most widely adopted sphincter-preserving procedure for ultra-low rectal tumors (2). However, when the tumor’s lower edge is located less than 2 cm from the dentate line, the stapling required to anastomose the lower rectum may damage or resect the dentate line, which is a critical structure for maintaining anal continence. Studies have shown that preservation of the dentate line is essential for satisfactory postoperative anal function, as it contains abundant sensory nerve endings that contribute to the rectoanal inhibitory reflex and discrimination of stool consistency (3). Studies have reported that about 30% to 50% of patients develop LARS after this procedure (4). While this technique could be used to preserve the anatomical integrity of the anus, the functional outcomes are often significantly reduced. To mitigate this risk, prophylactic ileostomy or small bowel stenting is generally employed. About 15–28% of patients with ultralow rectal anastomoses experience anastomotic leakage if fecal diversion is not performed. Ileostomy has long been the standard approach following LAR due to its technical simplicity and proven efficacy in reducing both leak rates and the need for reoperation. However, it carries stoma-related complications and requires a subsequent closure procedure, imposing additional physiological stress and financial burden on patients. An alternative approach involves the use of a biodegradable ileal stent (5). The stent is deployed in the terminal ileum to occlude the lumen, while a decompression catheter is placed proximally to the stent for fecal diversion. When compared with ileostomy, this approach avoids stoma-related complications and eliminates the need for reversal surgery, without increasing the risk of severe postoperative complications.

#### 3.3.3 ISR

ISR was first proposed by Braun and Schiessel in the early 1990s and represents a major advancement in anal sphincter-preserving rectal surgery. This surgical procedure is indicated when the tumor’s lower edge is at or below the levator ani plane, thus necessitating intersphincteric dissection to achieve a 1–2 cm distal resection margin (6, 7). The abdominal phase follows the standard LARs approach with dissection extending toward the dentate line. However, in the transanal phase, the distal resection margin is marked at about 1 to 2 cm below the tumor’s inferior pole. Electrocautery is then used to sequentially incise the mucosa, submucosa, and internal sphincter muscle. The rectum and mesorectum are subsequently externalized through the anus. After confirming the proximal margin, the rectal specimen is resected, and a coloanal anastomosis is performed. Compared to LAR, ISR allows resection of tumors located very close to the dentate line. However, the procedure carries notable disadvantages. Partial or complete resection of the internal anal sphincter and the inferior location of the anastomotic position increase the risk of LARS, which can severely impair quality of life (8). In some cases, persistent LARS symptoms may necessitate permanent stoma creation, often secondary to anastomotic leak, stricture, or complete sphincter dysfunction.

#### 3.3.4 Modified Bacon and Parks surgery

The Modified Bacon and Parks Surgery is primarily indicated for low rectal cancer located within 2 cm of the anus that cannot be directly resected and anastomosed via traditional laparoscopic or open abdominal approaches. This procedure involves transanal resection of the rectum, thereby avoiding the traditional Miles operation, which would require a permanent colostomy. Several sphincter-preserving techniques have been developed to optimize tumor clearance while maintaining anal function, among which the Modified Bacon and Modified Parks procedures are widely utilized.

In the Modified Bacon Procedure (9), the abdominal phase follows the standard LAR protocol. The perineal mucosa is circumferentially incised 0.5 cm above the dentate line. Subsequently, a dissection is performed cephalad through the submucosal plane to the superior border of the anorectal ring. The rectum is then transected at the anorectal ring, and the sigmoid colon is divided at least 10 cm proximally to the tumor. A screw-threaded tube is inserted into the proximal colon and secured with a purse-string suture 0.5 cm from its end. The tube, along with the attached colon, is then pulled through the anal canal, and the proximal colonic wall is anastomosed to the mucosa and muscle layers at the dentate line. The exposed tube segment is fixed to the perianal skin with bilateral sutures. This procedure preserves the dentate line and anal transitional zone, maintaining sensory function and continence. The screw-threaded stent diverts fecal flow, allowing the distal bowel to undergo necrosis and sloughing while the proximal colon adheres and heals to the anal canal and pelvic sidewall. This eliminates the need for a second-stage procedure to remove the extracorporeal colon, improving surgical safety and postoperative quality of life. However, although the Modified Bacon Procedure avoids the risks of anastomotic leakage, it can increase the risk of developing LARS, ischemic necrosis of the terminal colon, and anal stenosis (10).

In the Modified Parks Procedure, the perineal surgeon first designates a resection margin at about 1 to 2 cm distal to the tumor. A vertical incision is made through the mucosa and internal sphincter, followed by cephalad dissection in the intersphincteric plane until connection with the pelvic cavity is achieved. The colon is then externalized transanally. After mobilizing the mesentery, the colon is cut (transected) at least 10 cm above the tumor to ensure a safe margin and allow proper removal of nearby lymph nodes. The reconstruction is carried out using absorbable sutures in a single-layer interrupted fashion, creating an anastomosis between the colonic stump and the dentate line. This suturing technique incorporates a segment of the internal sphincter to ensure a secure connection. Compared with the Modified Bacon Procedure, the Parks technique carries a higher risk of anastomotic leakage and is associated with a greater incidence of postoperative bowel dysfunction (11).

#### 3.3.5 TaTME

TaTME is a bottom-up surgical procedure for rectal tumors and total mesorectal excision (TME) performed using either the TEM or TAMIS platforms. During the laparoscopic phase, the mesorectum is dissected up to the pelvic floor following standard TME principles. In the transanal phase, the rectal lumen is irrigated with povidone-iodine, and the surgical platform is inserted after adequate anal dilation. Anal retractors are used to facilitate visualization, and a full-thickness circumferential section of the internal sphincter is performed at about 1 to 2 cm distally from the tumor margin. The internal sphincter is partially or completely resected as required, and then the severed ends are sutured. The pelvic floor is then accessed through the intersphincteric space and pelvic hiatus, where a double purse-string suture is placed at about 1 to 2 cm below the tumor margin for lumen closure and tumor isolation. A full-thickness circumferential incision distal to the purse-string suture is performed to allow entry into the pelvic floor. Following a posterior-lateral-anterior dissection sequence, the mesorectal envelope is mobilized cranially along the holy plane between the visceral and parietal pelvic fascia until it meets the abdominal dissection plane, completing the TME. After adequate anal dilation, the rectal specimen is exteriorized transanally, the proximal sigmoid colon is transected, and the specimen is removed. Anastomosis is then performed using a transanal transection and single-stapled technique (TTSS).

This approach provides direct access to the perirectal planes of the lower mesorectum, enabling precise dissection of the tumor, adequate circumferential resection margins, and distal excision of the mesorectum. TaTME has been associated with favorable short- and long-term outcomes with reported anastomotic leakage rates ranging from 6.4% to 17.0% (12, 13). However, the incidence of LARS compared to standard laparoscopic TME varied across studies. A multicenter study by the European Society of Coloproctology demonstrated that patients with low anastomoses and male patients had a significantly higher risk of developing LARS (14). In addition, Bjoern et al. (15) observed no significant difference in the LARS scores between TaTME and laparoscopic TME.

#### 3.3.6 NOSES-PPS

**Figure 1:**
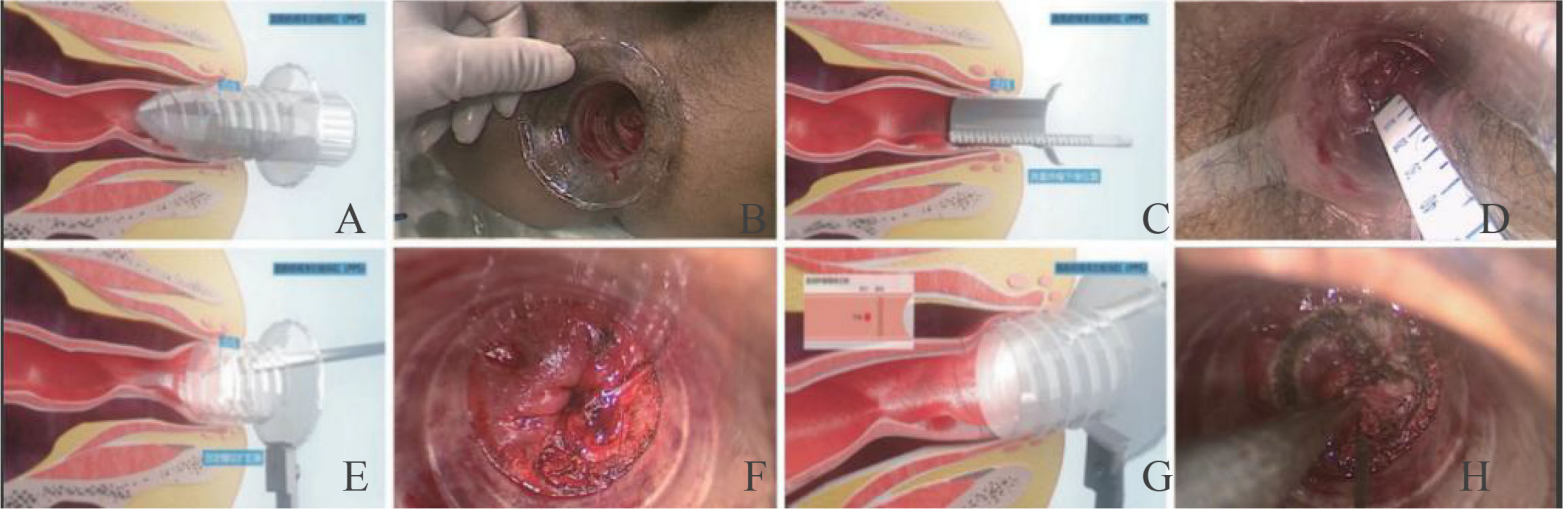
NOSES-PPS surgical procedure. **(A, B)** Insertion of a transparent threaded anal dilator. **(C, D)** Distance measurements from the lower edge of the tumor to the dentate line. **(E, F)** Determination of the resection margins under direct vision through the anal dilator. **(G, H)** Transsection of the rectum using an electrocautery device. Source: Chinese Journal of Gastrointestinal Surgery (2024), 27(11):1100-1106 [10.3760/cma.j.cn441530-20240828-00295], used under the CC BY-NC-ND 4.0 license.

The NOSES-PPS technique was developed by Liu et al. at Shanghai Tenth People’s Hospital, affiliated with China Tongji University, in 2018. This surgical (16) approach builds upon conventional laparoscopic techniques for low rectal cancer and is described in more detail in **Figure 1**. The surgical devices used for the procedure include a custom transparent threaded anoscopes, graduated anal dilators, tumor depth measurement devices, precision circular stapler anvils, and related accessories. The customized transparent screw-threaded anoscope and matching device are used to perform a transanal direct-vision resection of the distal tumor margin. Digestive tract reconstruction is then accomplished via a dual-technique anastomosis combining circular staplers with hand-sewn suturing. This novel surgical approach facilitates nerve-sparing sphincter preservation in ultra-low rectal cancer. The abdominal portion of the procedure is similar to that used for the LAR. The perianal phase involves the following steps: After anal dilation, a transparent threaded anal dilator of appropriate size is inserted. Under direct visualization, the tumor’s location, morphology, distal margin, and relationship to the dentate line are reconfirmed. The distal resection line is designed to ensure a complete resection with a minimum distance of 1 cm from the tumor. If the margin status is uncertain, intraoperative frozen section analysis is performed (17–20). The principle of “precision resection” is applied to maximally preserve the bowel wall opposite the tumor. High-frequency electrocautery is used to circumferentially mark the distal resection line on the rectal mucosa, followed by perpendicular transection while maintaining appropriate traction to avoid oblique cuts that could compromise margin integrity. The distal resection margin is sent for intraoperative frozen section analysis to confirm negativity. The tumor and mesenteric stump are then extracted through the anal dilator.

For the tumor resection, the mesorectum is dissected and trimmed to at least 10 cm proximal to the tumor. After skeletonizing the bowel, the rectum is transected, and the tumor specimen is removed. The anvil of a circular stapler is introduced into the proximal rectal stump, and the bowel is reduced through the anus. The proximal bowel is secured to the levator ani fascia with 4 to 6 sutures placed approximately 0.5 cm above the anvil rim, ensuring proper positioning to minimize anastomotic tension and restore levator ani traction on the rectum. A full-thickness purse-string suture is placed at the distal margin, incorporating both mucosal and seromuscular layers. The washer attachment of a precision circular stapler is threaded onto the anvil shaft and secured centrally to replace most of the resectable distal bowel segment and maximize the preservation of the normal distal rectal wall. Following anastomosis, the staple line integrity is confirmed, and circumferential reinforcement is performed with interrupted sutures. A rectal drainage tube is left in place for 5 to 7 days to reduce intraluminal pressure.

This technique optimizes the operative field and working space in the anal region, allowing precise bowel transection and reconstruction. Studies have shown that NOSES-PPS significantly improves sphincter preservation rates and postoperative anal function in ultra-low rectal cancer patients compared to conventional sphincter-preserving methods (21). Additionally, it also significantly reduces the need for prophylactic stomas(12% vs 37.3%, P = 0.015)and postoperative complication rates (12% vs 32.7%; P = 0.039) (22).

## 4 Discussion

Ultra-low rectal tumors are particularly challenging to excise using traditional endoscopic resection methods because of their proximity to the anal sphincter. During the procedure, surgeons must balance achieving clear surgical margins with preserving sphincter function. To address this issue, several advanced surgical techniques have been developed to improve surgical resection without compromising functional outcomes.

Established surgical techniques, including LAR, ISR, TaTME, modified Bacon, and Parks procedures, are widely practiced and have reached technical maturity. These approaches generally yield favorable outcomes in terms of tumor control and long-term survival rates. However, despite these advances, a significant proportion of patients continue to experience postoperative bowel dysfunction, manifesting as frequent bowel movements, urgency, incontinence, and irregular defecation. Such functional impairments can substantially affect daily life, leading to malnutrition, perianal dermatological issues, sleep disturbances, and psychological or social challenges, including anxiety, depression, and reduced work capacity.

Recent innovations, particularly the NOSES-PPS technique, have shown encouraging results in mitigating functional complications by enhancing sphincter preservation and minimizing surgical trauma to the distal rectum. Early evidence suggests that NOSES-PPS may improve postoperative bowel function, reduce complication rates, and enhance quality of life. A key strength of this approach is its potential to optimize functional outcomes and minimize postoperative morbidity. However, current studies are limited by small sample sizes and restricted application, and the findings remain preliminary. Large-scale, multicenter prospective trials are needed to validate the efficacy, reproducibility, and long-term functional benefits of NOSES-PPS across diverse patient populations. Currently, definitive conclusions regarding its superiority over established sphincter-preserving techniques cannot be drawn from the available literature.

In addition to the surgical techniques described above, the evolution of minimally invasive platforms has profoundly influenced the landscape of sphincter-preserving surgery for ultra-low rectal cancer. Robotic surgery, in particular, has emerged as a transformative technology in colorectal surgery. By providing three-dimensional high-definition visualization, articulated instruments with seven degrees of freedom, and enhanced ergonomics, robotic systems enable precise dissection within the confined pelvic space. The enhanced magnification capability of the robotic surgical system enables precise dissection along the microanatomical plane between the fascia propria of rectum (FPR) and the prehepogastric nerve fascia sheath. By performing intermembrane micro-space dissection between the FPR and the prehypogastric nerve fascia sheath, the technology can preserve the autonomic nerve main trunks, branches, and vascular supply of autonomic nerves while minimizing bleeding and surgical trauma.Several studies have demonstrated that robotic-assisted low anterior resection and intersphincteric resection are associated with improved postoperative functional outcomes, including lower rates of urinary retention and better anal continence, compared with conventional laparoscopic approaches (23). The precision afforded by robotic platforms aligns closely with the core principle of functional sphincter preservation: maximizing oncological safety while minimizing collateral damage to neural and muscular structures essential for continence. Therefore, the integration of robotic technology with advanced sphincter-preserving techniques represents a promising direction for further improving outcomes in ultra-low rectal cancer patients.

In addition to the choice of surgical technique, several other factors significantly influence the development and severity of postoperative LARS.Neoadjuvant chemoradiotherapy has been consistently shown to impair anorectal function through mechanisms including radiation-induced fibrosis of the pelvic floor muscles and sphincter complex, damage to the pelvic autonomic nerves. These effects can exacerbate LARS symptoms independent of the surgical approach (24, 25). Furthermore, the timing of ileostomy closure represents another critical determinant of functional outcomes. Prolonged diversion leads to disuse atrophy of the neorectum and sphincter complex, resulting in reduced reservoir capacity and impaired sensory function upon restoration of bowel continuity. Some studies have suggested that earlier ileostomy closure (within 3–6 months) may be associated with better functional recovery (26), though optimal timing remains debated.

Importantly, in the studies reviewed comparing different sphincter-preserving techniques, these confounding factors were not consistently reported or controlled for. As a result, it remains challenging to isolate the independent effect of surgical technique on LARS outcomes. Future comparative studies should standardize the reporting of neoadjuvant treatment protocols (including radiation dose, timing, and response) and ileostomy closure intervals to enable more rigorous comparisons of functional outcomes across surgical approaches.

## 5 Conclusion

Surgical excision of lower rectal tumors aims to fully excise the tumor, reduce the risk of local and distant metastasis, while preserving the anal sphincter to reduce the risk of fecal incontinence. Sphincter-preserving surgery for ultra-low rectal cancer has evolved from conventional open procedures to minimally invasive techniques. Recent advances, including three-dimensional laparoscopy and robotic-assisted platforms, have further enhanced the precision, safety, and feasibility of these procedures. However, surgical outcomes depend not only on the operative approach but also on comprehensive preoperative assessment, careful patient selection, and meticulous postoperative management.
